# From Touch to Mental Imagery: The Embodied Aesthetic Experience of Late-Blind People Engaged in the Tactile Exploration of Enrico Castellani’s Pseudo-Braille Surface

**DOI:** 10.1007/s11013-025-09904-9

**Published:** 2025-03-10

**Authors:** S. Uboldi, A. Bortolotti, G. Candeloro, A. Marasco, F. Sardella, M. Tartari, P. L. Sacco

**Affiliations:** 1 Department of Maternal-Child and Adult Medical and Surgical Sciences, University of Modena and Reggio Emilia, Modena, Italy; 2 Department of Neuroscience, Imaging and Clinical Studies, University of Chieti-Pescara, Chieti, Italy; 3 ISPC-CNR, Naples, Italy; 4 Enrico Castellani Archive, Milan, Italy; 5 metaLAB (at) Harvard, Cambridge, MA USA

**Keywords:** Mental imagery, Blindness, Embodied aesthetic experience, Enrico Castellani, Emotional Well-being, Haptic Visuality

## Abstract

This paper examines the embodied aesthetic experiences of late-blind individuals during tactile engagements with Enrico Castellani’s Pseudo-Braille Surface artwork. The study applies a mixed computational-qualitative approach, utilizing the Atlas-Ti software for semantic analysis of interviews with 21 participants. Categories emerging from the analysis suggest a vivid relationship between touch, mental imagery, emotional well-being, and the creation of meaning. Key findings demonstrate a transformation from a traditional pedagogical approach to an immersive aesthetic experience, marked by a significant meta-cognitive shift, transitioning from practical understanding to haptic contemplation and narrative digression. Sometimes, participants initially experience negative well-being due to difficulties in interpreting tactile stimuli, but this evolves into positive well-being as they engage in an imaginative process, invoking autobiographical memories and personal narratives. The study reveals that this personal and relational encounter with original art enables participants to overcome initial feelings of inadequacy, unlock creative freedom, and attain emotional well-being. The participants’ experiences are interpreted in the light of Walter Benjamin’s notion of Aura, unveiling the unique and authentic interaction between viewer and artwork in the realm of haptic perception. The results advocate for the inclusion of tactile aesthetics in art appreciation, emphasizing the potential for aesthetic experiences to contribute to the well-being and empowerment of visually impaired individuals.

## Introduction: Aesthetic experience and blindness—Enrico Castellani’s Pseudo-Braille Surface

In our over-visualized world, embracing any perspective other than the visual one is challenging for most people (Koca-Atabey et al., 2017). In the world of the blind, however, touch is a primary medium for relating to the world, an analytical sense that requires education to comprehend forms (Coster and Loots, 2004). The continuity and intensity of stimuli are essential for facilitating an aesthetic experience (Khaw and Freedberg, 2018), as substantiated by scientific research that demonstrates how blind individuals utilize other sensory modalities to construct an understanding of the world that is as rich and complex as that of sighted individuals (Harrar et al., 2018). Studies have shown that in blind individuals, tactile sensations play a pivotal role in various daily activities (Théoret et al., 2004). In all sensory modalities, tactile experience is considered the main channel of perception (Crollen et al., 2017; Phani Krishna et al., 2020).

Despite this, it is important to stress that blind people’s perception of the world is as concrete and objective as that of sighted people. The goal of this paper is to highlight the role played by mental imagery and embodiment in the construction of the reality of the world of blind people, along a path that moves from tactile exploration to verbal expression. The first part of the study provides an introduction to perception in the presence of blindness, followed by a definition of how tactile imagery is formed through mental imagery. Finally, we focus on the characteristics of aesthetic judgment. To this theoretical framework, we add the presentation of the case study of the semantic computational analysis of the narratives of a group of subjects with acquired blindness who ‘looked through their hands’ at an artwork by Enrico Castellani, one of the most prominent Italian artists of postwar 20th century.

This study adopts a mixed computational and qualitative approach to investigate the meaning-making processes related to blind people’s haptic vision experience of a contemporary work of art specifically intended for a blind audience. The notion of embodied interpretation is introduced to explore the complexity of the aesthetic experience mediated by haptic perception and translated by means of mental imagery and language symbolization and synesthesia.

**Fig. 1:**
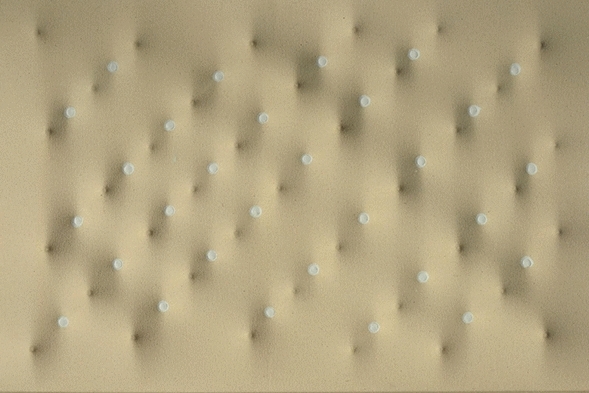
Enrico Castellani, *Pseudo Braille Surface*, 2014. Shaped canvas, 40.4 × 60.4 cm. Archive no. 14-007. Collection of the Blind and Visually Impaired Italian Union (U.I.C.I), courtesy of the Enrico Castellani Foundation. The work has been conceived by Enrico Castellani for the project *Art seen by us. The tactile image and its perceptive interpretation*, organized by the U.I.C.I., territorial section of Viterbo. The work was created without a pictorial finish to fit the sensory spectrum of the blind and visually impaired

The artwork that is the object of the study is *Pseudo Braille Surface* (2014), an original oeuvre by Enrico Castellani purposefully created to engage the sensory spectrum of blind people (Figure 1). Enrico Castellani, an internationally renowned artist, was born on 4 August 1930 in Castelmassa (Rovigo). He completed his middle school studies in Novara and Milan, attended the Brera Academy of Fine Arts and in 1952 moved to Brussels where he studied painting at the Académie Royale des Beaux-Arts and where in 1956 he obtained a degree in architecture from the Ecole Nationale Supérieure de la Cambre. In the same year, he returned to Milan and in 1959 with Piero Manzoni he founded the *Azimuth* magazine and the *Azimut* Gallery, making his debut in the artistic scene by launching both a conceptual and a physical platform (the magazine and the gallery, respectively). In 1959, he created the first of a series of relief surfaces, fleshing out a poetics that would be his permanent, rigorous stylistic signature, moving into an innovative sphere of serial repetition and fully exploiting the unlimited combinatorial possibilities of numerical structures. Since then, his artistic practice has evolved in many directions: extroflection, monochrome surfaces, relief, and the themes of time, rhythm, and space (Wirz Castellani and Sardella, 2023).

In a strict adherence to his method and aesthetics, and in fact leveraging upon the deep specificities of his artistic language, which blends in a unique and immediately recognizable way two seemingly distant techniques such as painting and relief, in 2014 he created the *Pseudo-Braille Surface*, in the context of a project that focused on the tactile dimension of aesthetic experience launched by the Blind and Visually Impaired Association (U.I.C.I.) of Viterbo, Italy. In this respect, Castellani himself remarked that

“The tactile impact with the surface of this painting is alienating and elicits very different reactions. The uniformity of the elements of the composition does not suggest anything that evokes previous memories. There is nothing that represents elements of a contingent reality, but this is the meaning of my work. Instead, reading the explicit reactions of blind people, precisely as such, is a universe of imaginative considerations and psychological introspections and searches for a value beyond the first tactile impact” (U.I.C.I. Viterbo 2018, p. 9).

Our analysis shows that the aesthetic experience of engaging with Castellani’s oeuvre had a significant impact on many levels:

(i) visual imagination processes sparked by the tactile experience;
(ii) psycho-emotional well-being;
(iii) changes in the mode of experience, from a traditional to a creative approach.

Moreover, the analysis of the experience points to an innovative re-interpretation of Walter Benjamin’s notion of *Aura* which provides a powerful scaffolding of the value sphere and meaning of the experience, as well as of its subjective well-being implications. The data suggest a strong relation between embodied interpretation and emotional well-being, and these results may enrich the heuristic framework for the understanding of the specific impacts of arts and culture on well-being. In particular, well-being relates through imagination to distinct features of the aesthetic experience flow. This analysis also invites a reflection on the potential of the tactile approach in the exploration and re-materialization of the artwork, an extremely relevant issue in the current digital era that demands a substantial adaptation of optical visuality to accommodate the new challenges posed by virtual epistemologies. An interesting relation is thus established with the growing body of studies and projects that use digital technologies to support blind and partially sighted people in experiencing cultural heritage (Bavi and Gupta, 2022; Muscarà and Sani, 2019). Advanced virtual reality technologies provide unprecedented opportunities for creating accessible heritage experiences, as in the case of the exhibition “Touching Masterpieces” (https://www.youtube.com/watch?v=ZukE86YTvhk) hosted in 2018 at the National Gallery of Prague, that allowed visually impaired visitors to touch the 3D models of some famous sculptures, such as the bust of Nefertiti, through haptic virtual reality gloves. Also due to the potential of application of digital technologies, the last years witnessed the rise of a lively debate on the design and evaluation of art and cultural heritage experiences for accessibility, to which this study contributes through its in-depth insights into the embodied aesthetic experiences of blind people.

In a nutshell, the central argument of this paper focuses on how tactile aesthetic experience in late-blind individuals unlocks a transformational process that moves from functional touch to aesthetic appreciation through the mediation of mental imagery and embodied interpretation. While the study engages with multiple interconnected themes—including synesthesia, emotional well-being, and aesthetic judgment—these elements converge around a core thesis: that meaningful aesthetic experience in our late-blind subjects emerges through a meta-cognitive shift from purely functional tactile exploration to what might be termed “aesthetic touch,” enabled by the dynamic interplay between haptic perception, mental imagery, and autobiographical memory. This transition is not merely a change in perceptual strategy. It represents a fundamental shift in the mode of engagement with art, one that generates positive well-being precisely because it liberates touch from its purely practical function to become a medium of aesthetic contemplation and personal meaning-making. The paper demonstrates how this process is particularly evident in late-blind individuals, who can draw upon both visual memories and tactile competencies to create rich aesthetic experiences. This framework allows us to understand how various elements—from synesthetic associations to emotional responses—are not separate phenomena but integrated aspects of the transformational process, all contributing to what we might call an “embodied aesthetic journey” from functional to contemplative touch.

## Literature Review

The literature provides ample documentation of the interests and desires of blind people to visit museums and appreciate the visual arts (Argyropoulos and Kanari, 2015; Buyurgan, 2009; Kozue et al., 2010; Hayhoe, 2013). It also reports how artistic education for the blind provides advantages in the appreciation of visual arts, allowing the access to important cultural information, promoting aesthetic enjoyment, and stimulating personal development, for example in the ability to think critically, refined through the analysis and interpretation of works of art (De Coster and Loots, 2004; Grum and Grum, 2016). The visual dimension represents a critical sphere for blind people, due to the long-entrenched (Rodas, 2003) power relationships implied by the hypervisibility/invisibility that accompanies blindness in societies mostly comprised of sighted individuals (Hammer, 2012, 2016b). Therefore, finding a way to acquire agency and voice in a sphere of experience related to the visual may become a considerable form of empowerment for blind people. Engagement with the visual arts can therefore benefit blind people in many respects, including not only social validation, but also enhancement of specific sensorimotor skills such as tactile ones (Kirby and D’Angiulli, 2011) as well as socialization skills (McMath, 2005). Moreover, art education can be the basis of cooperative learning paths that allow the encounter with art in groups, generating self-awareness and self-confidence (Axel and Levent, 2003). The recent study by Minghzhe et al. (2023) which conducted in-depth interviews on a group of 15 people with visual disabilities highlighted how blind users believe that the experience of enjoying art can be a valuable learning opportunity, and a source of encouragement and inspiration, of particular significance in the difficult moment when subjects need to psychologically bounce back after loss of vision. Some blind people construe their experience value in terms of activism, focusing upon accessibility rights of blind people (Kudlick and Luby, 2019). Furthermore, the experience of encountering art is identified as useful for experiencing well-being through positive emotional states, such as relaxation and enjoyment (Manship & Hatzidimitriadou, 2015).

In this section, we will review the existing evidence in some detail, with special attention to the perceptual and cognitive foundations of the experience of visual arts by blind people, in two dedicated subsections.

### The Embodiment Process: Haptic Visuality in Art Exploration

Understanding how visually impaired individuals perceive art is extremely relevant for a variety of reasons. In our societal organization, access to information and resources is vastly facilitated by visual perception, which remains the dominant sense from an attentive and perceptual standpoint (Adams and Kveraga, 2015). Although blind individuals all around the world have been passionately pursuing amateur drawing, sculpture, and photography for quite some time (Kennedy and Juricevic), their artistic endeavors often still encounter skepticism. Research in this area has shown that blind individuals can have rich experiences of visual arts (Hayhoe, 2017). Art allows the expression of important ideas, emotions, and beliefs in a multitude of forms, and profoundly influences human society (Dissanayake, 2001). Experiencing art can bring spiritual fulfillment and self-enrichment to individuals, and those in the blind community are obviously not an exception (Kleege, 2017). However, most art is experienced visually, thus creating access barriers for the visually impaired population (Candlin, 2003). Despite these challenges, blind individuals have found ways to engage with visual artwork (Hayhoe, 2017), and recognizing their unique perspective on art can be invaluable for various practical applications (Lisney et al., 2013). In today’s society where access to information and resources is heavily reliant on visual perception, understanding how these individuals experience art provides a unique perspective on multimodal perception (Ottink et al., 2022), and consequently on its reprocessing in terms of aesthetic preference (Lauwrens, 2019). The senses of sight, hearing, smell, and touch are frequently stimulated simultaneously and interact seamlessly (Gallace, 2013; Spence and Gallace, 2008), but touch provides a closer, more sensuous, and deeper intelligence of reality compared to vision (Classen, ; Montagu, 1971). While sight is a syncretic sense that grasps the world through the ‘wholeness’ of the visual field, touch proceeds through the exploration of spatial and temporal portions (Martin, 1992). Through this analytical procedure, seeing with one’s hands fleshes out an image through high-level mental operations (Paterson, 2017). Full possession of the skill of looking with one’s hands is the result of prolonged and constant education.

In *The Roots of Power*, Sheets-Johnstone (1994) provides a compelling analysis of the intricate relationship between tactile and visual experience, grounding her discussion in the fundamental nature of animate form. She argues that our tactile-kinesthetic experiences provide the foundation from which visual perception emerges and develops. Rather than treating vision and touch as separate channels of perception, Sheets-Johnstone demonstrates how they are deeply intertwined in experiential corporeal-kinetic form, that is, as the human foundational bodily way of knowing the world. Specifically, our primal animation is tactile-kinesthetic, suggesting that even visual perception is rooted in and shaped by our tactile-kinesthetic experiences (Sheets-Johnstone, 2011). This perspective is particularly relevant when considering the aesthetic dimension, as it suggests that what we perceive visually is always already informed by our tactile knowledge of the world. For Sheets-Johnstone (2016), this is not merely about cross-modal perception but about the very nature of animate existence: our capacity to move and touch precedes and shapes our capacity to see, creating a form of corporeal consciousness, a bodily way of knowing that undergirds all other forms of perception and understanding. This framework offers valuable insights for understanding how late-blind individuals might draw upon their prior visual experiences through the medium of touch, suggesting that the translation between visual and tactile modalities occurs at a deep, corporeal level.

In order to see through touch, the mind must build a complex schema of objects to stabilize their cognitive representation in memory (Bottini et al., 2016). The process of tactile exploration and placement of the part (space-time portion) in the overall mental image unfolds according to a haptic synthesis scanning process (Afonso et al., 2010). Even the overall image is the result of high-level cognition (Lahav and Mioduser, 2008), and as with visual images, tactile images also need to be linked to patterns of lived experience to be effectively memorized (Papadopoulos et al., 2017).

Research in this area has predominantly focused on how visually perceived qualities impact people’s liking or attraction to objects (e.g., Bar and Neta, 2006; Leder et al., 2011), leaving a relative gap in our understanding of how non-visual senses, such as touch or sound, might influence aesthetic preferences (Etzi et al., 2014). Indeed, the tactile perception in blind individuals does not possess the immediacy of visual perception. It is always mediated, mutable, and constructed over time (Heller, 1991). The form that emerges from tactile exploration is conceptual, a result of a sequential reconstruction of the object, perception after perception (Goldreich and Kanics, 2003). In the context of aesthetic enjoyment, a blind person does not contemplate the form, but rather the cognitive construction of a form induced by the imagination, a process akin to poetry (Pearce et al., 2016). The synesthetic properties of language play a crucial role in the development of blind individuals, fostering the growth of analogical and imaginative abilities (Buck-Morss, 1992). For instance, a congenitally blind individual reported that numbers, letters, months, and days of the week each have a precise position in mental space and a specific tactile texture (Bottini et al., 2022). This suggests that the absence of visual calibration impacts motor-sensory and inter-sensory integration during movement, diminishing the reliability of tactile signals in blind individuals. It is well known that, in blind people, the visual cortex takes on higher cognitive functions, including language (Tomasello et al., 2019). This functional reorganization at the neuronal circuit level provides further evidence of the complex relationship between sensory perception, cognitive processing, and language in blind individuals (Tomasello et al., 2019), all of which contribute to the definition of blind visuality.

Blind visuality is an embodied construct (Hahamy et al., 2021) that reflects not only socio-cultural factors affecting sensory perception (Rodas, 2009), but also the sense of self and its projections in defining one’s agency in the knowledge and political spheres of the social space of relationships with other subjects (Porkertová, 2022). It includes the concept of haptic visuality (Marks, 2000; Joy and Sherry, 2003) which is a process that makes viewers more active, even in the absence of blindness or low vision, because it requires drawing on one’s imagination to fill in what is not said or not finished in the image through a more fully embodied engagement. Haptic visuality consists of touching to see through an act that involves not only the hand but the entire body (Harland and Donnelly, 2020). Touch requires proximity so that sensation can flow from the point of contact to the rest of the body (Gepshtein et al., 2005), and the image arises from the act of touching or moving over the surface of the object so that the shape becomes definable, focusing on the structure and texture of the object. In the case study presented in this article, we specifically focus on the immersed aesthetic journey of individuals with visual impairments, engaged in the tactile exploration of an artwork. In this distinctive process, art transcends the confines of sight and transforms into a ‘symphony of touch,’ where hands and fingers become the instruments that craft mental imagery and evoke profound emotions and memories.

Haptic indeed is “the sensibility of the individual to the world adjacent to body by use of body” (Gibson, 1966, p. 97). If vision is a distal sense, the haptic sense is proximal, it is contact with the body (Thompson, 2016). In this perspective, Argenton (2012) focuses on the role of haptic perception in enjoying sculptures and proposes a research approach to better understand aesthetic behavior in the context of the visual arts. While the psychology of art perception has primarily focused on visual aspects, there has been limited research on haptic aesthetic processing of artworks. However, the literature (Chatterjee, 2008; Szubielska, Niestorowicz 2020) highlights the significance of haptic perception in the arts and suggests that it can enhance the interactive experience and appreciation of artworks for everybody.

As individuals who are blind or visually impaired explore artworks through their sense of touch, they partake in a deeply sensory and intimate interaction. Each contour, texture, and shape weaves itself into their personal narrative, enabling them to construct a mental representation of the artwork (Hayhoe, 2013). It unfolds as a dialogue between the physicality of the artwork and the tactile sensitivity of the individual, where art is not merely observed but wholeheartedly embraced (Lauwrens, 2023). Within this sphere, the absence of visual distractions often sharpens the focus on the core essence of the artwork. The texture of a sculpture, the relief of a painting, or the intricacies of a textile piece transform from mere physical attributes into conduits for a multi-layered aesthetic experience. Emotions, stories, and interpretations emanate from their fingertips, forging a profound connection between the art and the individual (d’Evie, 2017). The haptic exploration of art by those with visual impairments introduces a fresh dimension to our comprehension of aesthetics. It underscores the potency of touch as a means of artistic engagement and reaffirms the imperative of establishing inclusive art spaces where individuals of all abilities can access and revel in the beauty and ingenuity that art bestows (Eardley et al, 2022). In this context, the term ‘embodied’ assumes a profound significance, serving as a poignant reminder that aesthetic experiences are not confined to intellectual or visual realms; they are deeply entrenched in our physical beings (Wright-Carr, 2019), moving from the recognition that the embodied dimension of experience is the foundational layer to make sense of the human cultural sphere (Csordas’ and Loots, 2002). This field of research, as the following literature review shows, is a cross-sectorial one and involves not only perception and imagery but also matters of collective orientation to social justice (Hayhoe, 2017). It not only can enhance our appreciation of art but also highlights the necessity of ensuring art’s accessibility to all, regardless of their sensory capacities.

### Mental Images, Memories, and Narratives

Haptic perception shapes mental imagery in relation with the embodiment processes. As Duval observes (1993), and as we are witnessing in the case study, blind people’s mental images are subjective, that is, they are closely linked to individual characteristics and experiences. Mental images can refer to objects and scenes seen before sight loss or perceived haptically in blindness or reconstructed from verbal descriptions. The adaptive strategies used in cases of visual impairment for mental images creation are different and based on the type of blindness, particularly in cases of congenital or late-onset blindness (Burton et al., 2002). In the presence of late blindness, as in the case of the sample chosen and analyzed in the present article, a reorganization of knowledge and adaptation to new methods of exploration are necessary (Dormal et al., 2016; Röder & Rösler, 2003; Wan et al., 2010) and are enacted through specific and often traditional educational strategies and methods. Those who have once seen, even if for a short period, retain a visual memory that must integrate with new cognitive methods (Dulin, 2008). Research shows that late-blind people create mental images with visual characteristics (Hollins, 1989), and so the compensation strategies of blind subjects seem to extend across a range of verbal and auditory modalities.

The non-congenitally blind person brings back the visual images they experienced. There is substantial evidence that blind participants show similar patterns in tasks requiring visuospatial imagery, although in some such tasks performance is lower or slower. In a study by Venlierde and Wanet-Defalque (2004), congenitally blind participants even performed better on tests based on two-dimensional representations than late-blind and sighted participants. These results indicate the presence of visuospatial imagination. Furthermore, there is evidence that the visual cortex continues to support mental imagery in the blind triggered by other senses, such as the sound of objects (Cattaneo et al., 2008). The mental images of blind people are the result of a generative process based on other forms of images fleshed out through touch, sound or semantic information (Cornoldi, Vecchi 2000; Cattaneo, Vecchi, 2011; Renzi et al., 2013; Connolly et al., 2007), which is particularly relevant for images associated with autobiographical memories.

Visual images are complex multimodal representations that integrate different sensory channels—language, emotion, and narratives—and are an integral part of personal memory (Vannucci et al., 2016). Empirically, several studies of sighted individuals have shown that visual images are almost always present when people remember past episodes (Greenberg and Knowlton, 2014; Aydin, 2018). Studies show that the retrieval of autobiographical memories is accompanied by an increase in auditory imaging in blind participants compared to sighted participants, and moreover, blind participants demonstrate greater narrative capacity than sighted participants, through the retrieving of autobiographies in a continuous narrative format rather than moments in the form of images (Tekcan et al., 2015).

When it comes to narrative, our brain processes connect and make use of visual, linguistic, and tactile information (Sanchez et al., 2015). For example, an accurate description of an object might cause our brain to create a mental image of that object and simultaneously evoke associated tactile sensations, such as texture, temperature or roughness. Our brains tend to create mental images of objects we have touched or manipulated (Zhang et al., 2004). These tactile mental images can influence our understanding and acquisition of language (Iftime and Varasteanu, 2013) when we read or listen to familiar objects’ descriptions. The relationship between mental images, words, and the sense of touch is an intriguing dimension of human perception and sense-making which leads to a deeper understanding of how our brains integrate sensory, linguistic, and cognitive information in the process of perception and communication (Burattini et al., 2020). Goldin-Mcadow (2003) argues that imagination can play a role in understanding metaphorical utterances and suggests that the mind follows a system of perceptual symbols. In the present paper, we conducted an analysis on a series of interviews involving individuals who are late blind, detailing their encounters with an original work of art and the mental imagery evoked through tactile engagement. Our primary aim in this analysis is precisely to explore the connections and effects that emerge from their narratives, while also seeking to identify a procedural framework outlining the unfolding of the haptic visual experience.

## Methodology

### Touch as an Analytical Sense

The conceptualization of touch as an analytical sense in blind experience requires moving beyond simplistic compensatory models to understand its role within a complex matrix of sensory and cognitive processes. As Titchosky et al. (2019) emphasize, blindness is not merely the absence of sight but rather a distinct way of being in the world that generates its own forms of knowledge and perception. Touch, within this context, emerges not as a mere substitute for vision but as a cognitive organ—a sophisticated system for gathering and processing information about the world (Paterson, 2017).

As the skin establishes an inevitable, profound and at times strained relationship with the very notion of the self (Lafrance, 2009), the development of touch as an analytical tool involves a complex process of situated perceptual learning and cognitive reorganization (Cattaneo and Vecchi, 2011). This process goes far beyond simple tactile sensitivity; it encompasses the development of sophisticated scanning strategies, the ability to integrate sequential tactile information into coherent mental representations, and the capacity to translate tactile data into meaningful spatial and structural understanding. Blind individuals develop highly specialized exploratory procedures that allow them to extract specific types of information through systematic touch patterns (Heller, 1991).

Rather than representing a uniform condition, blindness encompasses a spectrum of sensory experiences that varies significantly across individuals and contexts (Kleege, 2017). The cultivation of touch as an analytical sense occurs within this broader ecosystem of adaptive strategies, that develop to support navigation and understanding of the world. Blind individuals create personalized “perceptual maps” that integrate touch with other sensory inputs, including sound, proprioception, and kinesthetic awareness (Ungar, 2018).

The sophistication of haptic perception in blindness is further evidenced by the role of tactile experience (Wong et al., 2011)—the ability to make fine discriminations based on touch that may be unavailable or less developed in sighted individuals. This expertise develops through an active learning process where touch becomes increasingly refined and analytically powerful through sustained practice and conscious attention to tactile information.

### Choice of the Artwork

Enrico Castellani’s *Pseudo Braille Surface* (2014) is a shaped canvas measuring 40.4 × 60.4 cm that exemplifies the artist’s distinctive approach to surface manipulation. The work presents a systematic arrangement of protruding elements across its surface, created through Castellani’s characteristic technique of stretching canvas over a structured support with precisely positioned relief points. These elements form a regular grid pattern that, while evoking the tactile language of Braille, does not actually encode any readable text—hence the “pseudo” designation. The work was intentionally created without pictorial finish, presenting itself as a mere surface with a rhythmically articulated topography. The deliberate absence of color or pictorial finish characterizes the work as a haptic field, uniquely conceived for tactile exploration. The protrusions vary subtly in height and spacing, creating a complex topographical landscape that reveals different qualities when explored from various angles and directions. As Castellani himself noted, the uniformity of the compositional elements intentionally avoids representational references, focusing instead on a pure tactile syntax, a formal language rooted in the realm of touch rather than vision.

The selection of Castellani’s *Pseudo-Braille Surface* as the focus of this study was guided by several methodological and conceptual considerations. First, this work represents a rare instance where a prominent contemporary artist specifically created an original piece intended for tactile exploration by blind audiences (Candlin, 2006). Unlike adaptations or translations of existing visual works into tactile forms, this piece was conceptualized from its inception as a haptic artwork, challenging the visual-centric hierarchy that often dominates art production and appreciation (Kleege, 2017).

The decision to focus on a work by an established artist from the mainstream contemporary art world calls for a closer look. Our choice of Castellani’s piece should not be read as an endorsement of the traditional art system’s authority over blind aesthetics, but rather as an examination of potential bridges between different modes of artistic experience. As Mingzhe et al. (2023) emphasize, the inclusion of blind people in mainstream art discourse is not about adapting to sight-centric norms, but about recognizing and legitimizing diverse forms of aesthetic engagement. Castellani’s willingness to create specifically for tactile exploration represents what Hayhoe (2017) describes as a “two-way dialogue” between established artistic practices and blind aesthetics, paving the way to approaches that do not merely accommodate non-visual appreciation but actively challenge the visual hegemony in art. As Kudlick and Luby (2019) argue, such initiatives can serve as entry points for broader institutional change, promoting a form of access as activism. The artwork thus becomes a site for knowledge-making in blind assemblages (Porkertová, 2022), where the authority of aesthetic judgment is not predetermined by visual conventions but emerges through multiple modes of sensory engagement.

The work’s deliberate lack of pictorial finish and its emphasis on surface manipulation hints at a conception of artistic experience that is not merely adapted from visual to tactile but reconceptualized through non-visual modalities. As Castellani himself noted, the work intentionally avoids representational elements that might invoke prior visual memories, instead creating a tactile aesthetic—an artistic experience that exists primarily in the realm of touch (Classen, 2012).

Furthermore, the artwork’s connection to Braille, even in its “pseudo” form, hints at the complex relationship between tactile literacy and the social empowerment of blind culture (Kleege, 2006). In particular, by creating a “pseudo” rather than actual Braille surface, Castellani deliberately moves away from the utilitarian aspects of tactile communication toward the aesthetic possibilities of tactile perception advocated by Rodas (2009).

The piece’s relatively modest dimensions (40.4 × 60.4 cm) allow for comprehensive tactile exploration within a comfortable timeframe. This is a crucial consideration in haptic art appreciation—the ability to construct a complete mental representation of the work through touch (Hayhoe, 2017). This scale facilitates an intimate encounter with art (Howes, 2018), where the entire work can be experienced through deliberate tactile engagement.

The artwork’s systematic yet non-representational nature also provides an ideal context for studying the transition from purely functional tactile exploration to aesthetic appreciation, a key concern in contemporary discourse on blind aesthetics (d’Evie, 2017). This aspect particularly aligns with our research interest in how late-blind individuals navigate between learned functional approaches to touch and more open-ended aesthetic experiences.

Finally, the relatively simple, bi-modal nature of Castellani’s work—engaging primarily with tactile and visual perception—reflects a conscious methodological choice for this initial study. While multisensory artworks incorporating sound, smell, and other modalities offer rich possibilities for aesthetic engagement (Howes & Classen, 2013), starting with a work whose sensory structure is relatively simple allows us to more clearly trace the pathways between tactile exploration and mental imagery formation. In particular, understanding how aesthetic experience emerges even from limited sensory inputs can provide valuable baseline insights for future research into more complex multisensory art encounters. The artwork’s focused sensory palette reflects a controlled complexity approach to studying aesthetic experience—beginning with more straightforward sensory configurations before moving to more elaborate ones. Examining basic sensory translations in aesthetic experience can thus reveal fundamental patterns that might be harder to identify in more complex multisensory environments. Future studies examining artworks with richer sensory profiles will be crucial for developing a more comprehensive understanding of multisensory aesthetic experience in blind individuals.

### The Target Population of the Study: Late-Blind Individuals

The decision to focus our study on late-blind individuals reflects specific research considerations regarding the relationship between visual memory, tactile perception, and aesthetic experience. Late-blind individuals sit at a unique sensory borderland, having experienced both visual and non-visual modes of perception, which gives them specific sensory affordances which are not found in congenitally or early blind subjects (see e.g., Cattaneo et al., 2013; Karim et al., 2022). This distinctive position offers valuable insights into the transformation of aesthetic experience across different sensory modalities.

Moreover, research indicates that late-blind individuals develop specific compensatory strategies that differ from those of congenitally blind people. As Frasnelli et al. (2011) demonstrate, late-blind individuals often retain access to visual imagery and can integrate these stored visual memories with new tactile experiences, creating cross-modal mental representations. This capacity for multimodal integration is particularly relevant when studying aesthetic experiences that involve both tactile exploration and imaginative processes.

However, our focus on late-blind participants should not be interpreted as privileging visual experience or suggesting a hierarchy of aesthetic capability. Rather, late-blind individuals’ experiences offer unique insights into the plasticity of aesthetic perception and the complex interplay between memory, sensation, and meaning-making. The transition from sight to blindness, as Kleege (2017) notes, involves not just the loss of one perceptual mode but the active development of new ways of knowing and experiencing the world.

Late-blind individuals are thus endowed with a dual consciousness of visual memory and tactile perception. This duality can be particularly illuminating when studying aesthetic experiences that challenge traditional visually centered art appreciation. Late-blind individuals have a chance to develop sophisticated strategies for translating between visual memories and tactile sensations, offering valuable insights into how aesthetic experiences can be constructed through multiple sensory pathways.

The selection of late-blind participants also allows us to examine the reconfiguration of the sensory hierarchy that occurs after sight loss (Paterson, 2020). This process of sensory reorganization (Collignon et al., 2013) involves not just the enhancement of tactile perception, but also the development of new cognitive frameworks of potentially high relevance for the processing of aesthetic stimuli. Such transformations can therefore offer important insights about the flexibility of aesthetic appreciation and the non-visual dimensions of artistic experience.

It is important to note that this choice of participant group does not imply that the experiences of congenitally blind individuals are less valuable or relevant to understanding tactile aesthetics. Rather, different perceptual histories offer complementary perspectives on the nature of aesthetic experience. Future research examining the experiences of congenitally blind individuals with the same artwork and with other, more multimodal ones would provide valuable comparative insights and contribute to a more comprehensive understanding of tactile aesthetics.

### Well-Being

Well-being is a complex, multidimensional construct that encompasses both positive and negative dimensions of human experience. Rather than representing simple opposites, these dimensions interact in sophisticated ways that shape overall psychological and emotional states. As Ryff and Singer (2020) note, well-being emerges from the dynamic interplay between positive experiences (such as joy, satisfaction, and personal growth) and negative ones (including frustration, uncertainty, and challenge).

Defining well-being represents a significant challenge for researchers, as the concept encompasses multiple dimensions of human experience and has been approached from various theoretical perspectives. As Dodge et al. (2012) observe, while research in well-being has grown substantially in recent decades, the question of how it should be defined remains largely unresolved. This definitional uncertainty has led to what Forgeard et al. (2011) describe as “blurred and overly broad definitions of wellbeing” (p. 81). The difficulty stems partly from well-being’s intangible nature (Thomas, 2009), making it challenging to define and even harder to measure. This conceptual challenge has persistently engaged researchers’ attempts at definition and measurement (Pollard & Lee, 2003). Many attempts to capture its essence have focused on describing dimensions of well-being rather than providing comprehensive definitions (Christopher, 1999). Despite such definitional difficulties, well-being has become one of the most widely studied constructs in the psychological realm and its scope extends beyond mere happiness to encompass personal development, fulfillment, and contribution to the community, suggesting the need for a more nuanced understanding that captures both hedonic and eudaimonic aspects of human flourishing (Marks & Shah, 2004).

The positive-negative continuum of well-being states is in turn a very challenging aspect to analyze. The positive dimension of well-being extends beyond mere hedonic pleasure to encompass eudaimonic elements such as purpose, personal growth, and self-realization (Keyes, 2021). Positive well-being therefore emerges not just from pleasant experiences but from meaningful engagement with life’s challenges and opportunities (Tov et al., 2022). Negative well-being, conversely, should not be viewed simply as the absence of positive states. The actual positive-negative well-being dialectics is much more complex and nuanced than it can be imagined. For instance, negative experiences can serve as catalysts for growth and development, playing crucial roles in psychological adaptation and resilience (Lomas & Ivtzan, 2016; Southwick & Charney, 2018), and acknowledging how such experiences can contribute to overall well-being by stimulating grit and personal development.

There is therefore a productive tension between positive and negative well-being states. Optimal functioning often involves not the elimination of negative experiences but rather their integration into a broader pattern of growth and adaptation, as well-being frequently emerges from the successful navigation of challenging experiences rather than their absence (Wong, 2011).

The notion of well-being in the context of blindness studies requires careful application to avoid reductive or ableist interpretations. Well-being for blind individuals should not be measured against normative visual standards but understood within their own embodied experiences and life contexts. This approach aligns with crip theory (Hanebutt & Mueller, 2021), a conception that recognizes disability not as inherently negative but as a complex lived experience that generates its own forms of flourishing. The development of a conceptual framework for the well-being of visually impaired people in the context of crip theory is a significant theoretical challenge that will hopefully be taken up in future research. In the present context, we do not make use on any specific measurement scale for well-being and refer to a generic qualitative formulation of the construct that is useful for the analysis and the interpretation of our subjects’ verbal accounts of their experience with Castellani’s artwork.

### Aura and Haptic Perception

Walter Benjamin introduced the notion of aura in his essay *The Work of Art in the Age of Mechanical Reproduction* (1936). Aura refers to the unique and authentic quality that a work of art possesses when experienced in its original form, such as a painting or a sculpture. According to Benjamin, aura is tied to the artwork’s authenticity, historical significance, and the presence of the artist’s touch and intention. In an age of mechanical reproduction, such as photography and film, aura is powered down as these reproductions lack the original’s unique quality. Benjamin’s concept of aura and the haptic perception of artwork can be seen as complementary aspects within the value sphere of art appreciation. They enrich the meaning of the art experience and can contribute to a sense of self-perceived well-being by allowing viewers to engage with art on both intellectual and sensory levels.

While Benjamin’s notion of aura was fundamentally conceived within a visual paradigm, its conceptual richness offers unexpected potential for understanding the unique qualities of haptic aesthetic experience. We then propose that the concept can be meaningfully extended to the haptic sphere, where it acquires new dimensions that paradoxically reinforce rather than diminish its significance.

In haptic perception, aura manifests not through the distance that Benjamin considered essential to visual appreciation (“the unique phenomenon of distance, however close it may be”), but through an unprecedented intimacy of contact. This apparent contradiction reveals a deeper truth about aesthetic experience: the “distance” Benjamin spoke of is not necessarily physical but phenomenological—it is the recognition of the artwork’s otherness even in the moment of closest contact. In tactile exploration, this dialectic of proximity and distance becomes particularly acute, as the perceiver simultaneously experiences both immediate physical contact and the artwork’s resistance to complete appropriation.

The authenticity that Benjamin saw as central to aura finds new meaning in haptic perception. While visual reproduction might diminish the aura of images, tactile engagement with an original artwork creates a form of authenticity that cannot be reproduced—each touch is unique, each exploration personal and unrepeatable. As Classen (2012) argues in her analysis of tactile aesthetics, touch creates a form of knowledge that is inherently tied to presence and immediacy. This haptic aura, we might say, is not weakened but intensified by its resistance to technological reproduction.

The aura of an artwork, as defined by Benjamin, contributes to its meaning. The authenticity and historical value associated with an original work of art can add depth and significance to the viewer’s interpretation. Haptic perception enhances this meaning by allowing the viewer to physically engage with the artwork, shaping a more intimate connection. The tactile experience can reveal nuances and subtleties in the artwork that might not be apparent through visual inspection alone, further enriching the meaning. Within the value sphere of art appreciation, both aura and haptic perception play crucial roles. Aura emphasizes the historical and cultural value of the artwork, while haptic perception emphasizes the sensory and experiential value. Together, they contribute to a holistic appreciation of art, encompassing both intellectual and sensory dimensions, and contributing to a sense of well-being that can lead to a heightened state of mindfulness and self-awareness.

Moreover, what Benjamin identified as the artwork’s “historical testimony” takes on new significance in haptic perception. The physical encounter with an artwork’s materiality creates a direct connection to its history of creation and previous interactions. As demonstrated by Howes (2018), tactile engagement allows perceivers to participate in a material dialogue with the artwork’s history, one that is fundamentally different from visual appreciation.

This reconceptualization of aura in haptic terms aligns with what Marks (2002) terms “haptic visuality”—a way of seeing that functions more like touch, emphasizing the material presence of the image. In our context, we might speak of “haptic aura”—the unique quality of aesthetic experience that emerges through physical contact with an original artwork. This quality encompasses not only the artwork’s authenticity and historical significance but also the unrepeatable nature of each tactile encounter.

### Sample, Data, and Software

The sample for this study consists of 21 totally blind subjects (11 men, 10 women, age 17-79; average men age 54.8, average women age 58.4) who have been invited to participate in an experience of tactile exploration of Enrico Castellani’s *PseudoBraille* surface. All of them are affiliated with the Blind and Visually Impaired Association (U.I.C.I.) of Viterbo, Italy. All the participants had become blind in adulthood (from 17 years old onwards), and this has been the main selection criterion among Association affiliates. Moreover, none of them had a previous professional experience with art or a regular attendance of art museums. Their museum attendance is mainly related to visits to the Omero Tactile Museum in Ancona, Italy (a museum institution whose institutional mission contemplates engagement of blind audiences), and to occasional visits to other museums organized by the association. The experience was unstructured, and every participant was free to remain with the artwork for as long as they wished. There has been a wide range of durations across participants, from just a few minutes to half an hour.

All participants have been interviewed during the experience. The interviews were unstructured, and each participant was invited to describe their experiences in their own words. All the interviews have been recorded with the informed consent of the participants. In addition, three additional, one-hour in-depth interviews have been conducted with three of the participants, with the help of a blindness expert. The aim was understanding how such an experience could be usefully related to the pedagogical practices adopted by a regional institute focused on educational programs for blind people, also by means of tactile experiences in museums.

The analysis of the interviews has been conducted by identifying the significant semantic categories as emerging from participants’ feedback. The relationships among the semantic categories have been investigated by means of speech analysis with the Atlas-Ti semantic analysis software. Such mixed method approach offers valuable insights, through a multi-perspective focus that highlights personalized perspectives on the experience, on its aesthetic dimension, and on personal well-being (Paddon et al., 2014).

The Atlas-Ti software is used in qualitative analysis for its remarkable potential in detecting emergent meanings from data, overcoming the limitations of static approaches to the relationships between variables. The software facilitates the development of theoretical models firmly anchored to the text, allowing the different categories of analysis to interact with the meanings construed by the subjects in their discourse. It has proven extremely useful in the field of qualitative analysis of the structures of communication and digital traces of human interactions (Muhr, 1997). Atlas-Ti is based on a cognitive network model that allows the formal organization of data and the synthesis of results in terms of the heuristic macro-categories set by the researcher during the design phase. In our survey, an inductive approach was preferred, which involved the construction of a theory rooted in the text, moving from a first reading and decoding of the categories, followed by their redefinition through comparison with the theoretical literature. In this perspective, the decoding phase is key. Next, we build networks of associations or causal relations between categories, to be understood as tools for analytical investigation and as mental maps for planning and theoretical development. This procedure allows the researcher to seamlessly reflect on the relationships and meanings during the data collection and coding process.

The analyses conducted through Atlas-Ti yield qualitative–quantitative data that can be juxtaposed to the results of the preliminary (quantitative) phase and the qualitative analysis of the speech. The first step in the application of the mixed method (Grounded Theory) (Glaser and Strauss, 1967) through the software involves encoding the data by means of unique identifiers (codes), which are then used as markers in the data. In the second phase, the codes are grouped into categories based on similarity and themes. Atlas-Ti allows to relate emerging codes to each other in text-based categories and to measure the groundedness and density of the code. Groundedness refers to the number of citations linked to a code; density refers to the number of other codes linked to a marker code. The results of the analyses will then be delivered in the final report, with graphs and representations of the emerging categories, in relation to the theoretical models.

## Analyses and Discussion

Many of the points emerging from the literature also resurface in our analysis of the interviews of the blind participants to the experience with Castellani’s Pseudo-Braille surface. When asked by the researchers what made the art experience enjoyable, the late-blind participants mostly focused upon the process of imagination based on visual memory. The emerging categories from our analysis, as stimulated by the experience of tactile engagement with the work, point to evocation of mental images relating to the real world, but also to processes of narrative and aesthetic imagination, through metaphors, synesthesia, and personifications.

**Fig. 2:**
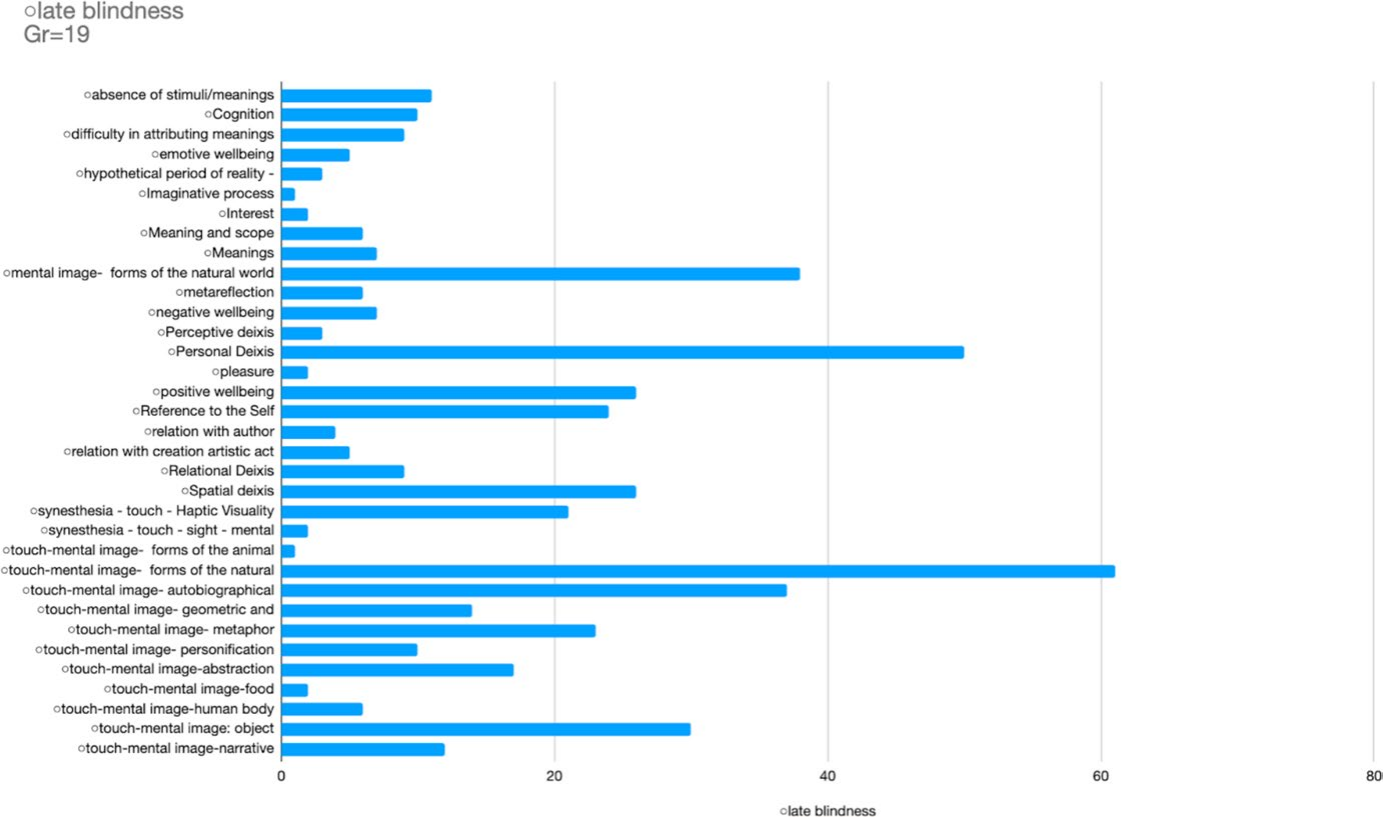
Atlas-Ti analysis of emerging categories

The most remarkable emerging categories (and the recurrence of sentences associated to each category) which have been found in our speech analysis of the interviews are the following (see Figure 2):

1. Touch-mental image—forms of the natural world (*gr* 61)
2. Personal deixis (*gr* 50)
3. Mental image—forms of the natural world—geography (*gr* 38)
4. Touch-mental image—autobiographical memory (*gr* 37)
5. Touch-mental image—object (*gr* 30)
6. Positive well-being (*gr* 26)
7. Touch-mental image—geometric and mathematical shapes (*gr* 14)
8. Touch-mental image—metaphor *(gr* 23)
9. Absence of stimuli/meanings (*gr* 11)
10. Cognition (*gr* 10)
11. Difficulty in attributing meanings (*gr* 9)
12. Relation with author (*gr* 13) and artistic act (*gr* 10).

The findings of the emerging categories analysis shed light on the intricate relationship between touch, memory, cognition, emotional responses, and artistic experience. The significance of personal narratives, diverse interpretations, and the role of sensory experiences in shaping aesthetic enjoyment emerges prominently throughout the analysis.

**Fig. 3:**
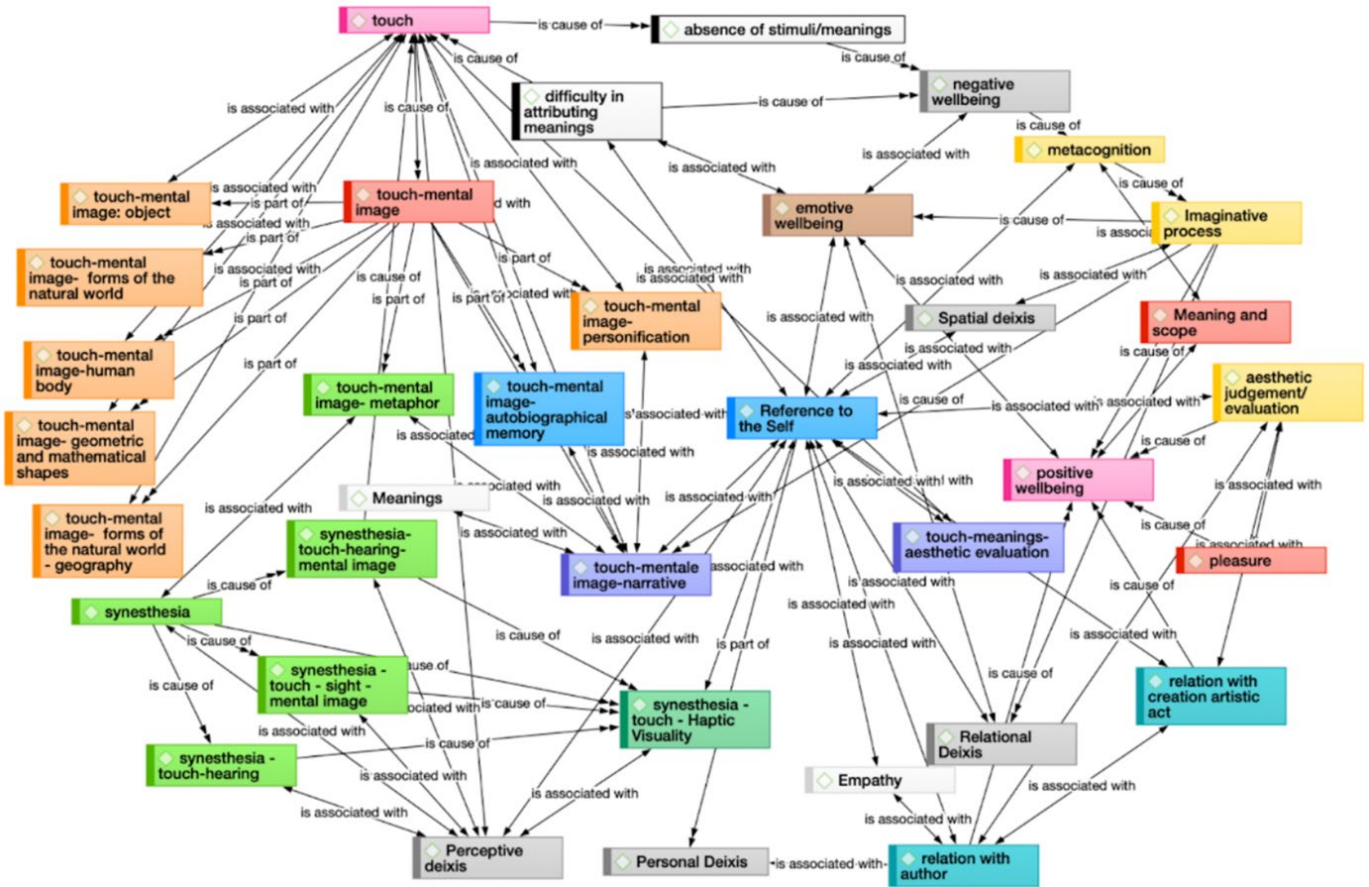
Meaningful relationships between codes (Atlas-Ti network)

**Fig. 4:**
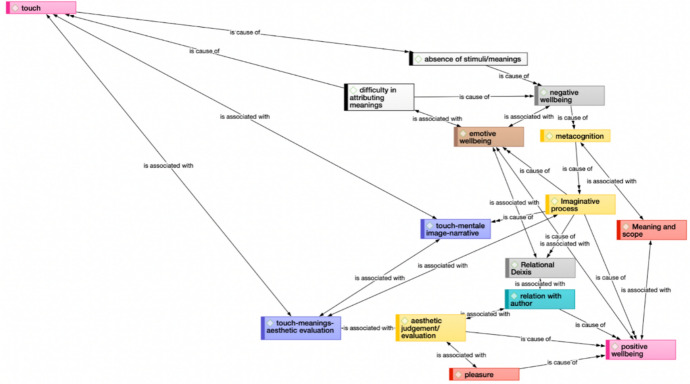
Processual pattern from touch to aesthetic experience, through meta-cognition shifting (Atlas-Ti network)

The following maps describe the most meaningful relationships between category codes (Fig. 3), which help us figure out the emerging patterns of meaning of the haptic process (Fig. 4).

Figures 3 and 4 provide a clear illustration of the complexity that arises from the personal narratives of the participants and clearly highlight that such experience has been on the one hand multi-layered and rich with many salient attributes, and on the other hand dynamic and transformational. Providing a verbal description of the properties of the graphs is difficult, but a few major features are worth being pointed out. One of them is the strong pathway from touch to mental imagery to perceptive deixis, showing how mental imagery plays a central role in situating the experience in space and time. Another is the crucial role of meta-cognition in the critical transition from an experience of negative well-being sparked by the lack of perception of fulfilling stimuli and meaning, to one of positive well-being activated by the imaginative process that results from the meta-cognitive shift, which further builds by creating/being reinforced by a sense of meaning and purpose. A complementary pathway instead moves through the uneasiness in attributing meaning to the experience to the effects on emotional well-being which in turn elicit positive well-being, with the reinforcement that emotional well-being exerts on relational deixis and aesthetic judgment. The situated experience of the relationship to the artwork, emotionally mediated, acquires an aesthetic character which becomes uplifting for the participant. And finally, another important pathway links touch to the reference to the self, which in turn establishes an empathetic relationship with the author. This again elicits positive well-being, both directly and through the mediation of aesthetic judgment. Even this brief and simplified account, that for the purpose of simplicity has been centered upon the well-being effects of the experience, is enough to appreciate the manifold complexity of the relationship with the artwork as resulting from the interviews. There is another important class of pathways that illustrate how the construct of haptic visuality emerges from mental imagery, synesthesia, reference to the self, and perceptive deixis, that can be reconstructed according to analogous lines of reasoning. Interestingly, haptic visuality and positive well-being are not directly related, but emerge as two key features of the experience whose connection is mostly mediated by the reference to the self and aesthetic judgment.

Figure 4 shows in more detail the parallel pathways that link the experience of touch and positive well-being specifically. We basically find three parallel pathways, going through meta-cognition, emotional well-being, and aesthetic evaluation. Meta-cognition mainly acts through the imaginative process and sense of meaning and scope, emotional well-being through relational deixis and the relationship with the author, and aesthetic judgment through pleasure.

In more general terms, a first striking feature that emerges from the analysis of the overall graph of the relationships between codes is the strong emphasis on the relation to the natural world. Such references reflect both the role of visual memory and the imprint of traditional educational methods for the blind, often involving tactile geographical maps and a strong focus on real-world exploration and movement. Moreover, they highlight how educational schemes influenced initial encounters with the artworks, shaping the cognitive processes involved in understanding the reality of the object. And in addition, they stress the significance of memory-based visual imagery in generating aesthetic enjoyment. Along the process, tactile exploration leads to visual imagination, culminating in verbal descriptions that refer to natural world and geographical frames, as well as to autobiographical and emotional memory tied to past moments. Such emotional memory intertwines with visual and sensory modalities, language, and narratives, fostering a creative phase of mental image construction based on haptically generated images.

Haptic visuality can therefore be defined in terms of somesthetic engagement through touch, and of the transition from touch to imaginative realms which involves a departure from the functional cognitive process toward lateral thinking, marked by effort, imagination, and occasionally frustration. There are significant individual differences in approaching art through these channels, which reflect varying levels of receptivity and inclination for exploration. Within this framework, emotional well-being appears linked to the ability to go beyond cognitive decoding for practical purposes and to embrace creativity, haptic contemplation, narrative digression, and remembrance guided by vivid mental imagery. Finally, the meaning of the experience is closely associated to the perception of the artwork’s authenticity value, which suggests a deep connection between the experience and the genuine nature of the artwork.

**Fig. 5:**
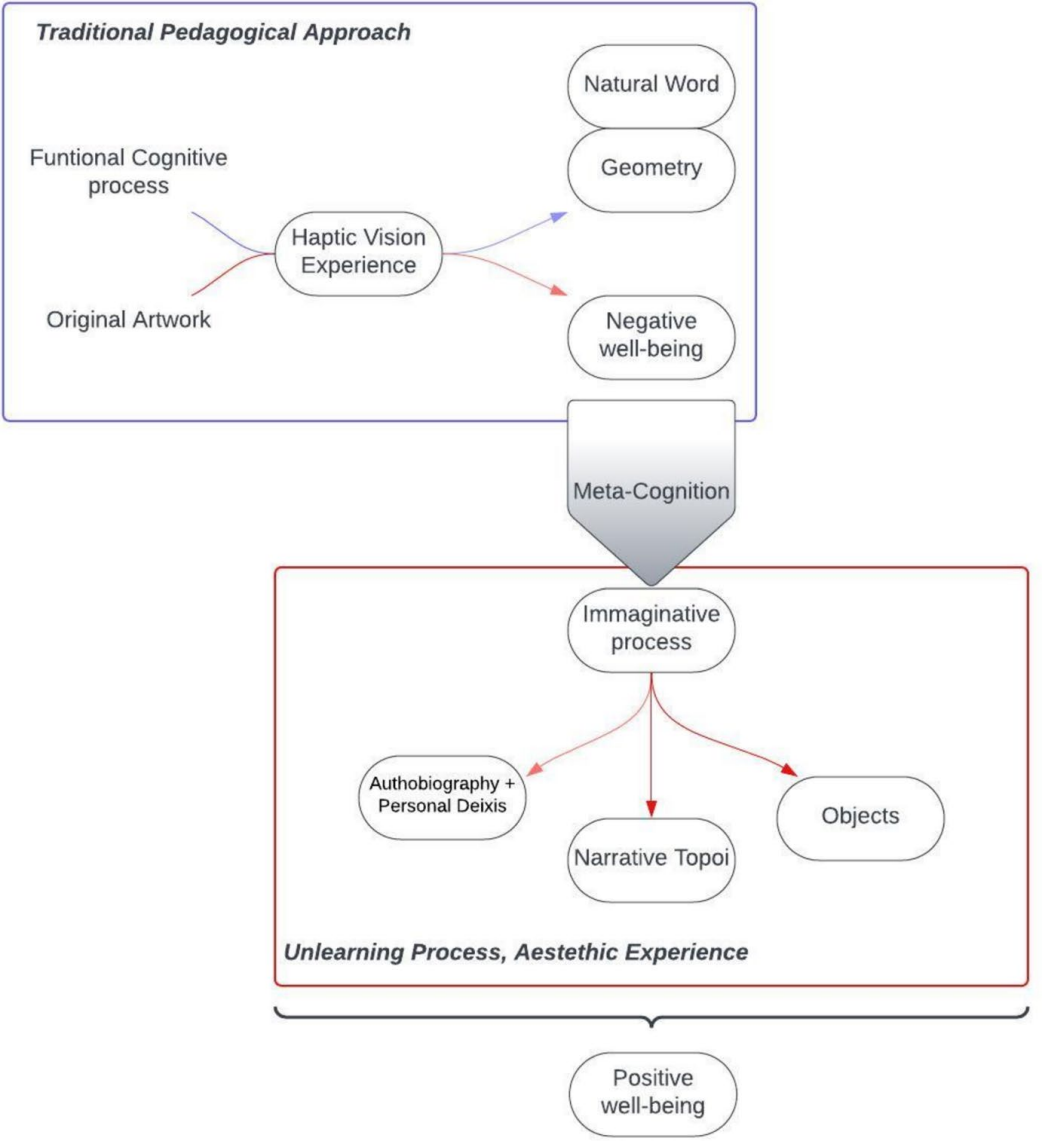
Transition from a traditional pedagogical approach to aesthetic experience, through a meta-cognitive shift

Another fundamental finding is that, through our comprehensive analyses, a discernible pattern in the haptic visuality process has emerged (Figure 5), fleshing out a crucial transition from the traditional pedagogical approach encountered by blind individuals—focused on comprehending the tangible attributes and functionalities of touched objects—to a state of aesthetic immersion and creativity. This transformational shift marks the substantial cognitive effort made by most participants, which chose to invest energy and mental resources to explore the potential of this unconventional approach. However, though embracing the endeavor, many participants expressed an initial sense of inadequacy and confusion (absence of stimuli; negative well-being), which however paved the way to a profound journey of discovery, self-narration, and imaginative exploration. In other words, for many participants, the experience was conceptualized as a journey from feeling inadequate to experiencing imaginative freedom, unleashing new opportunities for self-esteem, self-understanding, self-acceptance, resilience, creativity, and openness to experience. A trajectory that can be interpreted, among other things, in terms of a process of acknowledgment of self-efficacy and introspective comprehension, mainly pointing toward the domains of self-actualization and meta-cognitive awareness.

The experience of the transition is mostly narrated by participants as an imagination-driven voyage fueled by creativity, providing not only positive psychological impacts but also eliciting a remarkable sense of emotional well-being. This process of un-learning of a conventional, prescriptive approach primed by traditional educational methods in favor of aesthetic immersion can be seen, as already noted above, as a meta-cognitive shift. The meaning and purpose of learning is refocused, opening a possibility to get rid of the necessity to anchor the experience to cognitive constructs focused on the decoding of the world for merely practical purposes, to discover new psychological states of haptic contemplation, narrative digression and memory, through vivid mental imagery. By activating a range of responses and re-signification processes in the participants, the implications of experience for well-being are revealed through its capacity to unleash a sense of self-determination, freedom of choice, immersion in the experience, and empathic reaction. Haptic perception thus enriches the aesthetic encounter, revealing subtleties that elude visual inspection alone, and allowing physical engagement with the artwork that is conducive to a more intimate connection. This supports the idea that creative engagement demonstrates the transformational power of sensory experiences and their profound influence on emotional and cognitive states, highlighting the potential of such experiences in the promotion of well-being and self-discovery among visually impaired subjects.

In the following subsections, we can now get some deeper, more direct insights into the various steps of the experience from the participants’ own words, through excerpts from their interviews.

### Functional Cognitive Process

The groundedness analysis allows us to appreciate the prevalence of references that can be linked to the natural world and to geography, with their relations to visual memory on the one side, and on the other to traditional pedagogical models for blind people. Such models are aimed at a functional cognition of the object approached, by means of tactile geographical maps and constant references to the needs of movement in, and exploration of, the real world.

Roberto: “Does it have colors like this? Nothing? It seems to me like being in the middle of the mountains, but I can’t... It really seems to me like being, like when I could see a bit, in the middle of the mountains, maybe with some lakes. I imagine this, it’s something that relaxes me because I really like the mountains, and so I imagine all these not so high mountains. Maybe there’s a higher one above. I don’t know what else to say. Turning it vertically, it always seems like a mountain landscape to me; I feel like I’m in the middle of the mountains with the fresh air. I prefer the mountains to the sea, so I really imagine being here in the middle. When I could see a bit, I went there often, and so I really imagine them like this, with higher and lower mountains, and these spaces are lakes”.

Moreover, initial encounters with the artwork seem to be mediated by prescriptive models, shaping the cognitive processes involved in the understanding of the object’s physical reality. Additional perspectives from Maria Luz and Luigi highlight the diversity of evocative interpretations, with references to geographical features and, in Luigi’s case, a more unconventional analogy:

Maria Luz: “Turning it, I thought it would form a human body, but no”.

Luigi: “To me it looks like a constellation surface, in a geographical sense, with hills and valleys. I don’t see anything else specific because it’s more or less like that. If I can be a little explicit, they look like little girls’ breasts, but it doesn’t seem relevant to me”.

These insights are supported by an interview with an expert in blindness. Paola T., who (with all the limitations implied by the role of a sighted individual in the mediation of the experiences of blind people; see e.g., Hammer, 2013), emphasizes the ongoing debate within the community about what and how to teach, whether it is appropriate to educate for the development of perception through abstract thought, or to stick to a more functionally oriented education, targeting practical understanding of reality.

Paola T.: “So working on contemporary works, which are often abstract, is already complex, and it is something much discussed among those who deal with the world of the blind about what to teach, how to educate. There are old teachers who say: it takes us so long to teach about the real world, how can we then think of going to the abstract level [...] All education took place, for some things even much better than today, but it had a concrete focus”.

### Natural World, Geometry

As argued by Mingzhe et al. (2023), memory-based visual imagery is found to be significant for producing aesthetic enjoyment. For example, predominating semantic categories recall a process moving from tactile exploration, through visual imagination, and leading to the verbal restitution of frames corresponding to the natural world and geography. In this perspective, during the exploration of Castellani’s artwork, when perception is still linked to functional effort, it is evident that the first images that come to mind are nature-related. In this sense, Silvia too recognizes a landscape: ”It evokes a landscape, an Alpine landscape, seen from above, with the presence of some lakes” and Alessandro senses a mountain range: ”So there are things that look like mountains, but they are very schematic”. In this sense Paola T. suggests, in the aforementioned interview, that the link to geography may stem from a familiarity with tactile maps, extensively used in blind education for instructional purposes.

Within this functionally oriented educational framework, it is significant that the references to geometric and mathematical forms and rhythms are present, but to a much lesser extent than images of the real world.

Francesco: “You have to imagine everything. My mind goes to geometry; this is a rectangle”.

Bersa: “At the moment it’s a horizontal rectangle. I see sharp bumps; I don’t know if they have an order”.

Francesco: “It could also look like when you’re building a building and you put in different piles for the construction, the definitions, because it’s a geometric figure: these protrusions are six long and four high”.

Silvia: “They are symmetrical, no, that’s not true. Yes, then he put the highest points at the opposite ends, at the opposite corners of the rectangle, then comes the lower one, then the middle one, then there’s like, here, like many dunes chasing each other, but they’re pointed, not round, not rounded like dunes”. But there is this symmetry, the outer tips are mirrored and so are the lateral ones. It almost seems that there is a geometric pattern, that they have not been placed randomly”.

### Negative Well-Being

The passage from touch to a dimension that does not exclusively refer to the objective, tangible world, is marked by moments where difficulty, effort, in some cases even sense of inadequacy and frustration emerge. This amounts to shifting from a process of education of the blind person where every sensory and cognitive resource must be directed toward pragmatical tasks that improve security and competence, to one that rather involves an effort of imagination and abandonment to the aesthetic experience.

Francesco: “I think I said something stupid. I’m in trouble, I don’t know what else to do. It seems nonsense to me, I don’t know if I’m wrong”.

Irene: “The shape of a horse? The sky? No, I can’t, I’m sorry. Say that one doesn’t understand anything about it. Without seeing, I only feel many thorns”.

Maria Luz: “Nothing, there’s nothing else. I have no idea of the shape”.

This link between the experienced difficulty and negative well-being is confirmed by Paola T., who comments that the willingness to seek, or abandon oneself to, a given sensation varies across individuals depending on their unique backgrounds and learning experiences. In particular, she emphasizes the potential divergence in receptivity, so that individuals may not share the same inclination to actively explore or lose themselves in an artistic encounter. For those who have learnt to approach art with a concrete mindset, and thus looking for recognizable elements, their initial focus of attention may revolve around understanding the physical reality of the object, through a functionally oriented effort. If this is not successful, frustration may arise.

Paola T.: “So in this case we also need to understand who was benefiting from that work, what is the base from which it comes and the experiences they have had. And therefore it is not necessarily the case that there is the same willingness to seek or get lost in a sensation, because if a person has learned that they must seek the concrete features, that they must recognize something, their first approach is: “do I understand something?”, “no, I don’t understand” and so maybe they get nervous. If instead someone had taught that you can enjoy surfaces without necessarily finding anything other than: did the artist use a material that is pleasant to touch? Does this up-and-down of waves or curves create something positive or negative for you? There is the movement of the hand which is completely different from stroking a surface which is perhaps flat and different, does it give you different sensations?”

### Meta-cognition: One Step Beyond the Traditional Approach Toward Creativity

In the analyzed experience, blind people who manage to overcome this phase of difficulty enact a meta-cognitive shift toward a more creative way of learning and thinking. It shows how to activate a process of un-learning of their traditional approach, to explore categories linked to aesthetic enjoyment, pleasure, and emotional well-being with greater freedom. The narrativization process related to the onset of positive well-being is well documented in this passage from Susanna:

“As a first sensation, I feel some bumps. Then, however, it could also give the impression of two human figures, a man and a woman, even of two bears meeting in the forest. It could also be the meeting in the forest of two people who have lost their way, like Robin Hood, Robinson Crusoe. Either way, it also gives a sense of peace and calm. In this way, it gives even more the feeling of a forest, of animals, of squirrels climbing... It also seems like there’s a bit of wind, which gives the feeling that the leaves of the trees are being moved by the wind. If you turn it, it has a different effect. Actually, no, like this, it seems like a stormy sea, as if there were waves on an autumn day, with the leaden grey sky and this stormy sea. I said stormy things. It seems contradictory, but these stormy things give me peace, tranquility”.

### Imaginative Process: Autobiography, Narrative, and Objects

Once the dimension of negative well-being has been related to the un-learning of the functional approach, the imaginative process may begin. Rather than looking for abstract geometric patterns, participants now turn to autobiographical narratives, which are linked to both visual and emotional memory, and to the recollection of past moments. At this point, the creative phase kicks in, through a generative process of mental image construction building upon haptic imagery. This stage is particularly relevant for images associated to autobiographical memories, which are complex multimodal representations that integrate different sensory channels, language, emotions, and narratives (Cornoldi and Vecchi, ; Connolly et al., 2007). By addressing the artwork directly, through the personal and relational deixis of “you,” Carlo expresses a creative thought, a novel and associative form of thinking (Amabile, 1996; Stenberg, 1992; Fludernik, 2011):

“You give me the impression of a mountainous area... Remembering what I liked as a boy, a volcanic area. It reminds me of arid territories. A strange sensation… Even turning it vertically, I can’t grasp anything else. Touching it, I have a strange sensation. This reminds me of a stream; we used to go fishing there when I could see a little better”.

The creative process is thus linked to an experiential unconscious incubation (Poincarè, 1913). For instance, Olivana says

“I thought of a gallery near Termini station, where there was a graffiti that the painting reminded me of, but I don’t remember well what it was; I was fifteen. I see these flashes in my brain. (…) Now I was thinking of my mother’s heels, those shoes with heels that I liked so much and that I used to play with in front of the mirror... And so to the house where I lived when I was a little child, and the street near my house where the tram passed: all the memories of when I was a little child”.

References to the self and memory are marked by the cognitive anchors of personal deixis, with anachronies, shifts from first-person personal pronouns to plural pronouns (Stockwell, 2020). As in the case of Francesco, who moves from a first-person narrative to an “us” anchored in a re-enactment of his youth:

“It reminds me of when I was a kid and we used to go barefoot to swim in the ditch. There were all these prickly pear plants, and you had to jump because almost all of them were attached, and if you made a mistake, you ended up with your feet on the thorns”.

In some cases, the trajectory that moves from touch and through visual imagination reaches narration, proceeds through synesthesia to haptic visuality, eliciting the somesthetic dimension of touching with the entire body through the hand. In this perspective, Maria, Olivana, Rita, Silvia, and Elena recall passages between tactile, auditory, and visual sensory spheres:

Maria: “The idea of the sea, of lying on a mattress in the sea, the waves lapping gently against it. Nearby, the sand that slightly hides the small stones, and you search for them with your hand, slowly, in the damp sand near the shore. It’s a sensation I’ve been missing for many years, really... Touching it vertically, I like the idea of being on a small volcano all to myself and being able to touch the crater, the lava. It’s a beautiful feeling. The contrast between fire and water: the warmth of the sun, the fire, the sea, the water. It’s always the idea of the sand, of lying there stretched out”.

Olivana: “Now I start to think of sweets, ice cream, gummy sweets, liquorices in the shape of a little triangle that were there when I was little”.

Rita: “Mountain people singing, I don’t know why it gave me this feeling here”.

Silvia: “Maybe there’s a bit of wind, maybe there’s a bit of wind on an auditory level, maybe because it’s me making noise with my hands”.

Elena: “The only image I have of the night of San Lorenzo is from more than twenty years ago: we were by the sea, I don’t remember with whom, and there was this shady sky, I would dare say darker than cobalt blue... and yet it seemed that the sky was illuminated by a light coming from a very distant point. When you looked up, you saw this whole trail of stars, and you saw that it was full, so full that you couldn’t see the space between them. Each trail had its shapes, each group of stars, even if they were all piled up, but looking at them was like looking at the clouds, when it rains on the clouds and the clouds take on all kinds of shapes. When I put my hands on the painting, apart from the first three-seconds, it was like this”.

The process of imaginative construction can also make use of references to experience and objects in the world: “touch-mental image: object” is the category that best expresses the results of the aesthetic fruition process, because it contains the highest creative effort—in opposition to the functional, customarily learned cognitive process—toward a form of lateral thinking, as in the cases of Bersa, Francesco, Maria Luz:

Bersa: “It could remind me of boiling water or, I don’t know, a salt shaker, an egg holder”.

Francesco: “It could look like the Alberobello *trulli*. It could even look like the armor of a Roman, with all the spikes coming up”.

Maria Luz: “They could be chess pieces”.

Or, through more meaningful connotations, using a narrative grammar that is highly evocative also at the level of visual imagery, as in Elena’s words:

“For example, a field of Native American tents seen from above. These tents are scattered around the reservation, which can also be under high mountains, because there is this hilly stretch here, for example, which is not exactly... I mean, they are not lined up, as I feel them, but a little bit shifted, as if it were a mountain range, and below, on the left side, here and there, all the small tents of the Indians. And then there are these, I don’t know what to call them... as if they were the heads of nails or pins, as if they were many small wells, closer or further away, but all regular and in the middle of this city of tents. (...) On this side, positioned like this, it’s as if there was the feather of this Apache chief, as if this feather was flying towards the village”.

### Positive Well-Being and the Auratic Dimension

Furthermore, emotional well-being appears to be related to the possibility of abandoning the cognitive constructs of decoding the real world for practical purposes and abandoning oneself to states of haptic contemplation, narrative digression, remembrance, through vivid mental images.

This is the case of Irene who exemplifies the process from touch to the difficulty in assigning meanings to abandonment and imaginative digression guided by remembrance up to the emergence of the sensation of emotional well-being.

“I feel a canvas. I feel all the thorns the same. I would like to say many things, but I feel everything the same... Here, instead, it’s a little different: the figure of a little child... I thought I had felt something, a tree, a house... And instead I feel everything the same. How can I say: ‘I feel a tree’ when I don’t feel it, ‘I feel a house’ when I don’t feel it, ‘I feel the figure of a Madonna’ when I don’t feel it... I only feel thorns, and that’s it, and I can’t say anything else... These are the hands, the eyes are not there. I don’t feel anything, I don’t understand anything. The shape of a horse? The sky? No, I can’t, I’m sorry. Tell them that I don’t understand anything. Without seeing, I only feel many thorns. It could be a forest... It could be a castle, a stream... A forest with trees seen from above. I am convinced that I feel a figure. If I had felt something, I would have said so, for the hands of those who can’t see act as if they had eyes. But now I can only say what is in my mind: a castle, a stream, a panorama. It reminds me of when I was a child and went to the countryside and, just for fun, ended up in the middle of the brambles. It seems like yesterday; it was the time of the chestnuts; I ended up in the middle... Others laughed; I shouted because they didn’t pick me up. But at this moment I don’t feel any excitement. Again, it could be something sandy, from the Sahara. All sand. All spikes, all the same, some lower than others. A castle with a lawn... Many things, it could be... Blessed is the one who can see... Ah, it’s the same with you, I thought you could see with your eyes, and instead you’re like me. They could also be animals, horses, which I like so much. It could be the sun, or the evening sky with stars and the moon. A spring. It gives me a feeling of peace”.

According to the participants themselves, quoting what one participant said during one of the in-depth interviews, the pleasure stimulated by haptic exploration is explained by blind users as based on the imaginative power exercised through the direct engagement with an original work of art. During the interview, experienced meaning emerges as closely related to the authenticity value of the artwork:

“What remains of the experience of encountering the artwork? It’s a part of me, still inside me... maybe it was a discovery, an initiation, an awareness, each of us experienced it differently, some with irony or with more emotional involvement, maybe even too much for some of us... because it all depended on the moment each of us was living when there was contact with the artwork. Each of us encountered this artwork at a completely different moment in our lives, and therefore it brought out different states of mind. (...) Then we went to the Omero Museum, which has tactile works that allow you to get to know them, but Castellani’s work is different; it’s like an anti-stress. The work of art is original, it was built for us; it was there waiting for us, and it was you who built it by coming into contact with the work of art through your sensations, your words, your thoughts... it was something absolutely intimate... how can we say... the painting was yours in that moment because you activated it... so we had a very different experience when we touched the object, the statue in the museum, or when we touched Castellani’s work of art”.

Castellani’s work managed to activate a series of responses and re-signification processes in the participants. We could frame this process, as discussed above, in terms of a suitable re-adaptation of Walter Benjamin’s aura. The artwork became a subjectivity the participants could relate to, and not a mere object anymore, in the moment of the deepest aesthetic encounter, that of somesthesia.

In some cases, categories emerge from the analysis that point to the relationship with the author, as in the case of Marco:

“In the meantime, I think that the opportunity to touch a work by Castellani is an extraordinary thing. It’s such abstract art that sometimes it’s even difficult to visually understand the interpretation of the work. But I have the feeling that, to a certain extent... as far as I’m concerned, you have to imagine a little. Imagine, in particular, that this painting is struck by light which, depending on where it comes from, creates shadows. This painting, which seems to me to be rectangular, has irregularities which, thanks to the light, create shadows which, in turn, create visual interpretations, so it’s more difficult for us. It seems to me to be in a desert part of the world, with these dunes, more or less high. So I think of this dry area, the desert, as I said, or maybe the planet Mars, as we imagine it. Now, it may be that... in fact, I’m definitely off the track compared to what the artist wanted to represent with this work. I am still touched, because the more you touch this work, the more different sensations you discover. In this way you can imagine the infinity behind this work, also because infinity always has free interpretations, infinite interpretations of what this work can represent”.

Or in the case of Sandra, who traces the creation process through touch:

“I am very fascinated by the technique because I am searching for my artistic self and at the moment everything gives me ideas. It is these dimensions that can be related to the model of flow and surrender to the aesthetic experience capable of supporting well-being”.

Whereas Maria Rita introduces a meta-reflection, opening up to the emotional restitution of the experience.

“To touch a work of art is something special. It is even moving”.

These three testimonies reveal distinct but complementary aspects of tactile aesthetic experience that can be analyzed through different theoretical lenses. Through Kleege’s (2017) perspective on blindness and art, Marco’s testimony exemplifies tactile imagination, that is, the complex interplay between touch, memory, and creative visualization. When Marco describes imagining light and shadows while touching the work, he demonstrates the sophisticated capacity of late-blind individuals to integrate tactile information with visual concepts, even in approaching abstract art. His ability to move from immediate tactile sensations to elaborate mental landscapes (deserts, Mars) illustrates the translational competence of blind art perceivers. Classen’s (2012) anthropological approach to touch helps us understand Sandra’s testimony. Her fascination with technique and search for the artistic self reflects the idea of the “knowing hand,” characterizing touch as a mode of understanding that is simultaneously technical and deeply personal. Sandra’s experience articulates how tactile engagement with art isn’t merely receptive but actively creative, a tactile crafting of knowledge, and the reference to flow states hints at the transformational potential of touch in aesthetic experience. Maria Rita’s brief but profound observation, her description of touch as “something special” and “moving” suggests a kind of haptic sublimity, a moment in time when touch transcends its ordinary functional role to become a source of aesthetic wonder. The emotional transport of her response relates to an affective resonance of tactile encounters with art, where touch becomes a medium not just of perception but of emotional and existential significance (Paterson, 2007).

The dimensions linked to the relationship with the author and the work, the awareness of the artwork authenticity, and the auratic value are connected to the experience of significant aspects such as the abandonment to the aesthetic through imagination, which can be linked to the flow construct (Csikszentmihalyi, 2014; Nakamura & Csikszentmihalyi, 2002). Flow is characterized by a sense of self-determination and freedom of choice, by a transport (or immersion) in the experience, by empathic responses. Flow can also be understood as an evolutionarily designed coping resource against stressful situations. Recent studies highlight how it is associated to dopamine release, through the activation of the forebrain structure of the putamen, a recruitment of the empathy circuit, in particular the left inferior frontal gyrus; a reduction in activity related to self-referential processing, in the prefrontal cortex, and the activation of memorization processes. All this, accompanied by a decrease in the activity of the amygdala, induces a significant reduction in stress in subjects engaged with the experience (Riva et al, 2016; Ulrich et al., 2014).

The transportation metaphor is used during another in-depth interview:

Eufemia: “At the beginning, at least I took it a bit unconsciously, let’s go and explore, I thought... but when you’re there, the artwork imposes a concentration, an effort within you perhaps, and leads you to impressions. This artwork somehow brought us, dragged us into a very strong reflection... I don’t know why, but this transport was shared by forty people^1^. I heard some of my colleagues and friends who participated in this experience, and everyone was still carried away... Everyone brought out anything, for example, at that moment... I felt like a fighter against the world; I didn’t accept this certain situation [blindness] and so I wanted, by force, I had to do things... in each of us, in some way, brought out something very intimate”.

The value of the experience is returned as ‘therapeutic’ by another participant:

Paola: “At that time I was also experiencing the unbearable state of loss of sight, and everything came out, sadness and melancholy... Later, perhaps, anger was added, but at that moment, in front of Castellani’s work, there was more strength in me... The strength that comes from trying to stand, and when we did the public presentation of the artwork and Castellani’s painting was hanging on the wall, I approached it and said: How could this little thing bring all this out? Are you Pandora’s box? Because so many beautiful things, both positive and negative, came out... (...) We don’t know why this work of art made us dig, see and, above all, express certain things in an absolutely unpredictable way. For example, I had to go to the psychologist to accept various things, then I came here to have this experience and found myself saying things that, when I read them again... I said, how could I get them out like that, right? Because there was such a strong transport that made us go inside... go inside the artwork and come out with something of ourselves that was hidden... somehow it was maybe the most healing thing, if you want to put it that way. For some of us it was also the first opportunity to confront others and our disability. I call it Pandora’s box. (...) We had one member who brought up emotionally powerful experiences that she had had at the age of six, yes, and it was something that she would never have said, she would never have thought that she could come to this”.

These powerful testimonies relate to several theoretical frameworks that help us understand the therapeutic and transformative dimensions of tactile aesthetic experience. Eufemia’s testimony particularly resonates with Laura Marks’ (2000) approach to embodied memory. When Eufemia describes being “dragged into a very strong reflection,” she exemplifies the capacity of haptic experience to unlock embodied memories and emotional states that might otherwise remain inaccessible. The collective nature of this “transport” that she describes suggests how tactile experiences can create shared spaces of emotional recognition and transformation. Paola’s deeply moving testimony can be understood through Elisabeth Hsu’s (2005) work on the therapeutic potential of tactile engagement. The metaphor of Pandora’s box, and the way the artwork enabled the emergence of hidden emotional content demonstrates the excavatory potential of touch, bringing buried emotional experiences to consciousness. Moreover, the therapeutic value she finds in the experience demonstrates how emotional processing and psychological integration can be facilitated by an emotionally arousing physical contact. In addition, Paola’s reference to psychological therapy and the artwork’s role in facilitating unexpected emotional expression can be understood through the lens of Howes’ (2014, 2022) work as a form of sensory therapeutics. Paola’s engagement with material culture through non-visual senses enables new forms of self-understanding and emotional processing. Both testimonies also strongly resonate with Csordas’ and Loots (2002) somatic modes of attention and with the notion of embodied healing. The participants’ experiences can be seen as instances of embodied breakthrough moments where bodily engagement (driven by touch in this case) facilitates psychological and emotional watersheds. The transformation from resistance (“I didn’t accept this certain situation”) to acceptance and strength exemplifies how aesthetically meaningful physical engagement can lead to psychological integration.

## Conclusions

This study has focused on the haptic visuality process related to the experiences of visually impaired individuals engaging with an artwork originally created for a blind audience. It highlights the profound impact of sensory experiences, particularly tactile exploration and emotional memory, on the cognitive and emotional dimensions of the participants. Our main result is the characterization of the transition from traditional pedagogy to aesthetic immersion experienced by participants during their engagement with the artwork. Such transition has elicited a significant meta-cognitive shift toward an aesthetic experience that generates positive well-being, partially fueled by an initial negative well-being reaction linked to the difficulty and frustration in the early phase of engagement with the artwork. Through the process, the participants’ initial feelings of inadequacy have turned into a journey of imaginative freedom, self-discovery, and emotional well-being.

This transformational process, akin to self-efficacy and meta-cognitive awareness, marks a shift from decoding the physical world for merely practical purposes related to the limitation of familiar sight-related affordances deriving from the loss of sight, to embracing states of haptic contemplation, narrative digression, and vivid mental imagery. The meaningfulness of creative engagement and the related enhancement on the emotional and cognitive dimensions as well as the increased positive well-being among the visually impaired participants clearly emerges from their interviews, demonstrating the transformational power of direct engagement with original artworks and its potential for self-discovery after a major sensory deprivation. Our results highlight the intrinsic connection between creative engagement, sensory explorations, and their profound influence on emotional and cognitive well-being in the visually impaired population, shedding light on promising new avenues for enhanced self-discovery and empowerment through aesthetic experiences.

In conclusion, the transformational power of art not only holds the promise of greatly benefiting blind individuals by creating opportunities of knowledge acquisition, empowerment, (re-)construction of identity, and holistic well-being, among others, but also opens a real possibility to empower them in becoming demonstrators of multimodal aesthetic experiences. As pointed out by Classen (1998, p. 350), in Western culture the visual dimension has taken over most other forms of aesthetic expression, including dance and music where the visual element increasingly predominates over the kinesthetic and sonic ones. In this regard, the blind have much to say to sighted individuals as to how to regain a more balanced and sensuous engagement with all the dimensions of aesthetic experience (Hammer, 2016a). This perspective is of particular relevance in the context of a rethinking of cultural institutions such as the museum as experience spaces engaging all the senses and not just the visual, to the benefit of all kinds of visitors (Classen, 2017), and more generally of acknowledging disability as a distinguished and powerful form of imaginative agency which is valuable not just for disabled people but for society as a whole (Mills and Sanchez, 2023).

However, despite this very promising perspective, the accessibility of art to people with disabilities remains a critical yet often overlooked concern. With over 2.2 billion people worldwide facing vision problems, our societies, predominantly shaped around the centrality of visual experiences, more or less inadvertently exclude individuals who cannot see properly. This situation not only limits their engagement with artistic expressions but also disregards the well-being potential of tactile aesthetics, with significant social justice implications. This study provides a relevant contribution in this direction, which aligns with an evolved, broad conceptualization of accessibility to culture and heritage beyond the physical and perceptual aspects (Deffner et al., 2015). Our results emphasize the appropriation of culture/heritage by the visitor through personal connectedness, intertwining with their own story (narrative production) and own reproduction in a novel, subjective form.

Understanding and embracing the tactile exploration of artworks is pivotal in creating inclusive art experiences, not only for blind or low-vision individuals, but also for sighted ones, thanks to the disclosure of the uncharted realm of awe that is accessible through touch. Haptic exploration and embracing multiple sensory perceptions pave the way for a more inclusive embodied aesthetics and for a still largely unexplored avenue to cultural welfare that resonates with the diverse perspectives of all individuals in our society.

The main limitations of our study are the fact that it involves only one artwork, and the relatively limited size of the sample. Future studies covering several different artworks and involving participants with different histories of blindness could provide further important insights. For instance, it would be very interesting to compare the experience pathways of late-blind people with those of congenitally blind ones, and possibly also with those of low-vision individuals. It would also be interesting to compare the experiences of participants from different cultures, which typically integrate in different ways visual, auditory, and haptic elements in their cultural scaffolding of experiences. Getting a more comprehensive understanding of the effects of blind people’s engagement with the artworks could open up a new, exciting line of research and policy experimentation. This would also hopefully lead to a rethinking of the traditional educational models offered in the training of blind people, that tend to focus on practical concerns solely, therefore implicitly denying the importance and role of aesthetic experiences, among others, in their space of affordances. Relieving the psychological distress of blind people, largely related to the visual normativity of our societies, by recognizing both the right and the capacity to access profound aesthetic experiences may become an important pillar of a new public health approach to visual impairment, and possibly to other forms of impairment as well.

## Data Availability

All the interview materials are available from the authors upon request.

## Footnotes

1 The group of blind people accessing the experience was larger than that included in the study sample. Other people were not considered because they were not late-blind.
